# Evolution of chemically induced cracks in alkali feldspar: thermodynamic analysis

**DOI:** 10.1007/s00269-022-01183-9

**Published:** 2022-05-03

**Authors:** Rainer Abart, Elena Petrishcheva, Gerlinde Habler, Christoph Sutter, Franz Dieter Fischer, Jozef Predan, Marko Kegl, Franz G. Rammerstorfer

**Affiliations:** 1 Department of Lithospheric Research, University of Vienna, Josef-Holaubek-Platz 2, 1090 Vienna, Austria; 2 Institute of Mechanics, Montanuniversität Leoben, Franz-Josef-Strasse 18/II, 8700 Leoben, Styria Austria; 3 Faculty of Mechanical Engineering, University of Maribor, Smetanova ulica 17, 2000 Maribor, Slovenia; 4 Institute of Lightweight Design and Structural Biomechanics, Vienna University of Technology, TU Wien, Gumpendorfer Straße 7/Objekt 8, 1060 Vienna, Austria

**Keywords:** Chemically induced fracturing, Alkali feldspar, Crack spacing, Wavy cracks, Dissipation rate, Thermodynamic Extremal Principle

## Abstract

A system of edge cracks was applied to polished (010) surfaces of K-rich gem-quality alkali feldspar by diffusion-mediated cation exchange between oriented feldspar plates and a Na-rich NaCl–KCl salt melt. The cation exchange produced a Na-rich layer at and beneath the specimen surface, and the associated strongly anisotropic lattice contraction lead to a tensile stress state at the specimen surface, which induced fracturing. Cation exchange along the newly formed crack flanks produced Na-enriched diffusion halos around the cracks, and the associated lattice contraction and tensile stress state caused continuous crack growth. The cracks nucleated with non-uniform spacing on the sample surface and quickly attained nearly uniform spacing below the surface by systematic turning along their early propagation paths. In places, conspicuous wavy cracks oscillating several times before attaining their final position between the neighboring cracks were produced. It is shown that the evolution of irregularly spaced towards regularly spaced cracks including the systematic turning and wavyness along the early propagation paths maximizes the rate of free energy dissipation in every evolutionary stage of the system. Maximization of the dissipation rate is suggested as a criterion for selection of the most probable evolution path for a system undergoing chemically induced diffusion mediated fracturing in an anisotropic homogeneous brittle material.

**Supplementary Information:**

The online version contains supplementary material available at 10.1007/s00269-022-01183-9.

## Introduction

We employ gem quality alkali feldspar as a model system for studying fracturing due to anisotropic chemically induced lattice contraction in a homogeneous brittle material. The fracturing behaviour of alkali feldspar is of interest in different contexts. Alkali feldspar typically undergoes clastic deformation during tectonic movements in shallow levels of the Earth’s crust (Sibson 1980). Fracturing of feldspar is also important in technical applications such as drilling and blasting (Katsabanis 2020) and in mineral and rock comminution (Aditya et al. 2017). Moreover, due to its ubiquity on the Earth’s surface, alkali feldspar is also an abundant constituent of airborne mineral dust, and the crystal surfaces resulting from natural comminution by fracturing determine the ice nucleation activity of feldspar particles in clouds (Kiselev et al. 2017, 2021).

Fracturing of alkali feldspar single crystals was studied by means of indentation experiments, where *fracture toughness* was calculated from the length of induced radial cracks (Broz et al. 2006; Whitney et al. 2007). Apart from triggering by an external load, fracturing of alkali feldspar may also be induced by a heterogeneous eigenstrain state, which arises from the temperature and compositional dependence of its lattice parameters in combination with non uniform temperature or compositional fields within a feldspar crystal. Alkali feldspar is a framework silicate forming a binary solid-solution between the Na (NaAlSi$_3$O$_8$-albite), and K (KAlSi$_3$O$_8$-K-feldspar) end-members (Smith and Brown 1988). Its crystal structure is comprised of a three-dimensional framework of corner-sharing AlO$_4^{5-}$ and SiO$_4^{4-}$ tetrahedrons, and the alkali cations are located in large framework cavities. Depending on chemical composition, pressure, temperature, and the state of Al–Si ordering on the tetrahedral sub-lattice, alkali feldspar may have monoclinic C2/m or triclinic C1 symmetry (Ribbe 1983). The lattice parameters of alkali feldspar exhibit a considerable compositional dependence. In general, the *a*-, *b*-, and *c*-lattice parameters shrink with increasing Na-content, where the effect is substantially larger for the *a* parameter than for the *b* and *c* parameters (Kroll et al. 1986; Angel et al. 2012). This implies an anisotropic contraction of the crystal structure, when a K-rich alkali feldspar is shifted to a more Na-rich composition. In a *dry* environment, the rate of Na–K cation exchange between alkali feldspar and a NaCl–KCl salt melt is limited by the interdiffusion of Na and K on the alkali sub-lattice of the feldspar (Petrovic 1972; Schaeffer et al. 2014; Petrishcheva et al. 2014, 2020). A shift towards more Na-rich compositions by the in-diffusion of Na and the concomitant out-diffusion of K thus produces a Na-rich layer extending from the crystal surface into its interior, the thickness of which increases with time. The associated contraction of the crystal structure leads to a tensile stress state in the Na-rich surface layer with the largest tensile stress component approximately parallel to the crystallographic a axis. In gem-quality sanidine from Volkesfeld (Eifel Germany) and orthoclase from Madagaskar fracturing is induced, when the feldspar is shifted towards more Na-rich compositions by more than about 12 mole % corresponding to a maximum tensile stress component of about 300 MPa (Neusser et al. 2012). When cuboid plates of Volkesfeld sanidine or Madagaskar orthoclase with either the (001) or the (010) surfaces polished are exposed to cation exchange, parallel cracks with a characteristic spacing are produced. The crack spacing decreases with increasing extent of compositional shift and thus with increasing eigenstrain and corresponding tensile stress state (Scheidl et al. 2014). This is in line with theoretical predictions (Bazant and Ohtsubo 1977; Bazant et al. 1979; Sumi et al. 1980; Keer et al. 1979) and shows that the location and number density of the cracks emanating from polished felspar surfaces are primarily controlled by the applied chemical shift, whereas randomly distributed surface defects and surface roughness only play a subordinate role. After crack nucleation, the in-diffusion of Na from the newly formed crack flanks and the associated eigenstrain and stress states lead to crack growth at constant rate (Petrishcheva et al. 2019). More recently, a new approach for the numerical simulation of diffusion controlled crack growth and of the complexity that may arise from growth instability among multiple chemically induced parallel cracks emanating from polished feldspar surfaces was presented (Predan et al. 2020), who employed stability considerations that are common in structural mechanics. The theoretical and practical relevance of similar chemo mechanical fracturing was addressed by Wei et al. (2021).

Here we report new findings regarding the evolution of mode I edge cracks from chemically induced fracturing in alkali feldspar. We present statistical data on crack length and crack spacing as observed in time series experiments. In particular, we focus on the evolution of cracks from crack nucleation with non uniform spacing on the sample surface through an intermediate stage characterized by systematic turning and sometimes wavy crack morphologies to nearly uniformly spaced straight parallel cracks. We discuss maximization of the entropy production rate, or in other words, maximization of the dissipation rate as a criterion for identifying the most probable evolution path for a system undergoing chemically induced, diffusion mediated fracturing.

## Methods

Chemically induced fracturing in alkali feldspar was investigated experimentally by several authors (Neusser et al. 2012; Scheidl et al. 2014; Petrishcheva et al. 2019). Detailed descriptions of the experimental procedure can be found there and in the supplementary information. Only a brief summary is given here.

### Cation exchange experiment

**Fig. 1:**
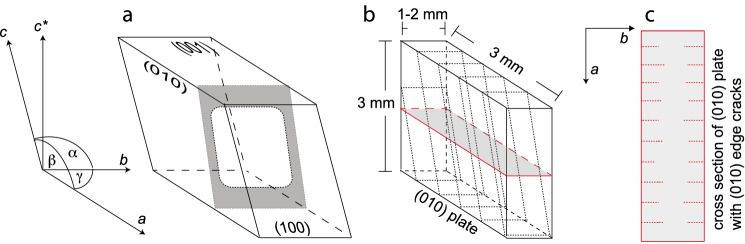
Schematic sketch of parallel cracks induced in (010) plates of Volkesfeld sanidine; **a** schematic crystal bounded by (100), (010) and (001) planes, the grey shaded area indicates an edge crack emanating from the (010) and (001) surfaces. **b** (010) plate, the dashed lines indicate traces of the cracks on the sample surface, and the shaded area indicates the position of the cross section shown in (**c**) with traces of (010) edge cracks (dashed lines)

Gem-quality sanidine from Volkesfeld (Eifel, Germany), which is transparent, colorless and free of inclusions, cracks or any other flaws (Neusser et al. 2012) was used as a starting material. The sanidine is monoclinic with space group C2/m and has a composition of $c_{\mathrm{K}} = 0.85$, where $c_{\mathrm{K}} = n_{\mathrm{K}} /\left( n_\mathrm{K} + n_{\mathrm{Na}} \right)$ is the atomic fraction of K on the alkali sub-lattice. The distribution of Al and Si in the tetrahedral framework is highly disordered with $\Sigma$t1 = 61 (Neusser et al. 2012). Cuboid plates with dimensions of 3 $\times$ 3 $\times$ 1–2 mm with the 3 $\times$ 3 mm surfaces oriented parallel to the (010) lattice plane were prepared using a diamond wire saw. The (010) surfaces were polished with diamond paste down to 0.25 $\upmu$m grain size. A schematic sketch of the sample geometry is shown in Fig. 1.

The crystal plates were subjected to cation exchange with an NaCl–KCl salt melt with a composition of $c_{\mathrm{KCl}} = 0.25$ at 850 $^\circ$C, where the composition of alkali feldspar in equilibrium with this salt melt is $c_{\mathrm{K}} = 0.50$. As a consequence, cation exchange produced a compositional shift from the original towards more Na-rich compositions. The salt was added in excess so that its composition remained essentially unchanged during cation exchange with the feldspar. After heat treatment for 2, 3, 5, 7, and 12 days the samples were quenched in cold water and the traces of the cracks were visible on the polished 3 $\times$ 3 mm surfaces (see supplementary information).

### Analysis of crack spacing and crack length

**Fig. 2:**
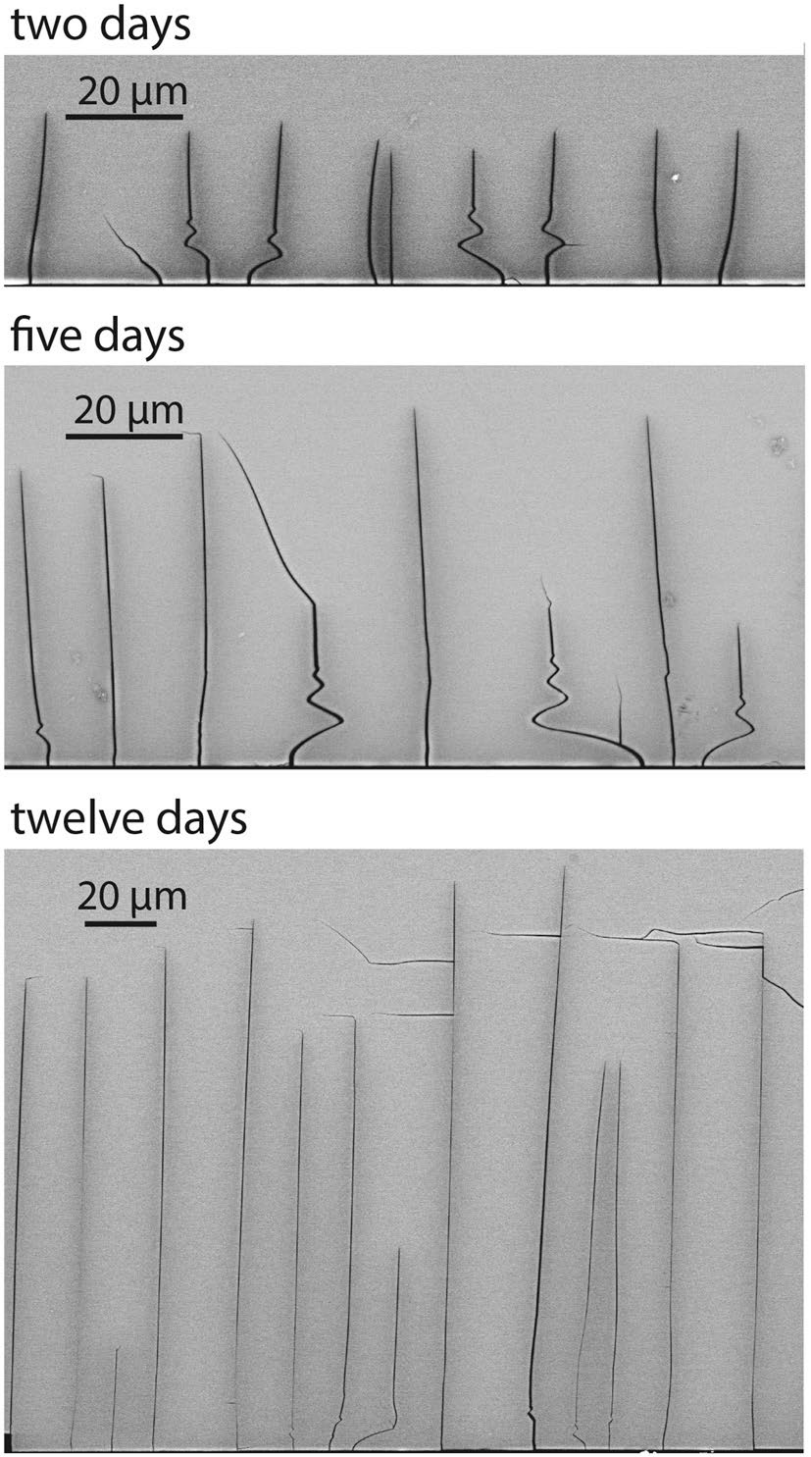
Exemplary BSE images of cracks emanating from a polished (010) surface (horizontal traces at the bottom of the images) of alkali feldspar after cation exchange for 2 days (top panel) 5 days (middle panel), and 12 days (bottom panel). The slightly darker grey shades along the cracks reflect a local enrichment in Na and depletion in K of the feldspar in the immediate vicinity of the cracks. The sub-horizontal cracks following the (010) cleavage of the feldspar and some other cracks are devoid of Na-rich halos indicating that they formed either during quenching or during preparation of the cross sections

Traces of parallel cracks emanating from the polished (010) surfaces are visible on the cross sections of all samples. The cracks enclose an angle of about 81$^\circ$ with the cross-sectional plane, so that the apparent crack spacings observed on the cross sections (Figs. 2, 5, and 7) need to be corrected by multiplying by $\cos 9^\circ = 0.9877$ to obtain the true spacing. For each sample between 160 and 240 cracks were documented on a series of Backscattered Electron (BSE) images. For each individual crack its length and the distances to the neighboring cracks were measured. The crack spacing was measured at 1, 3, 6, 11, 19, 28, 36, 44, 52, 61, 69 and 77 $\upmu$m below the polished (010) surface.

## Results

### Crack morphology

BSE images of the cracks obtained from the time series experiments are shown in Fig. 2. In all images, the trace of the polished (010) surface is horizontal and visible at the bottom of the image. The cracks extend nearly perpendicular to the polished (010) surface. This is due to the fact that the direction of the strongest contraction due to cation exchange and, thus, the largest tensile stress component lies in the (010) plane (Neusser et al. 2012). The cracks are oriented sub-perpendicular to this direction. The average crack length increase with time, but they show considerable scatter within each sample. Many cracks are curved in the first few $\upmu$m below the sample surface, and several cracks exhibit a ”damped oscillating” behavior. The relatively dark grey shades along the cracks correspond to chemical alteration halos, with elevated Na and and reduced K concentrations.

**Fig. 3:**
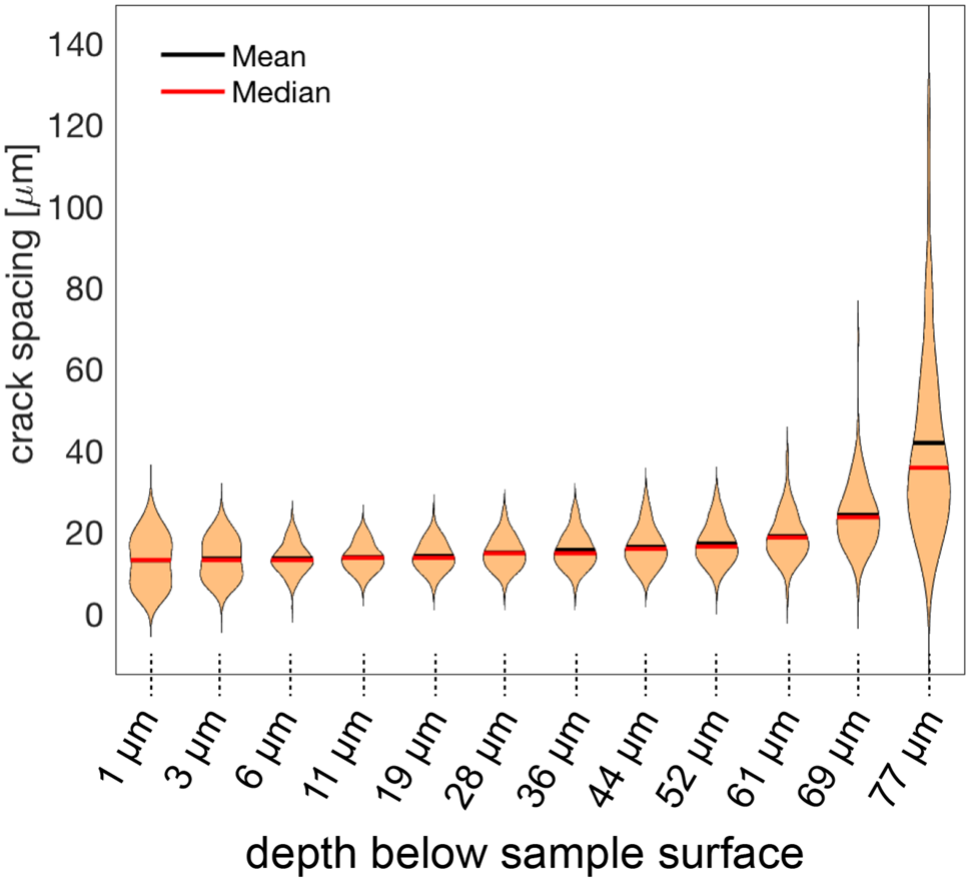
Violine plots showing the distribution of the crack spacings at different depth below the polished (010) surface of the sample; the data are from the 7 days experiment

For all considered run durations, the average spacing of the cracks is about 13.3 $\upmu$m on the sample surface. The distribution of the crack spacings changes with increasing depth below the sample surface. The crack spacings are rather irregular on the sample surface and become more uniform over the first about 20 $\upmu$m below the surface. In Fig. 3 the distribution of the crack spacings is shown for different depths. A histogram representation is given in the supplementary information. At 1 $\upmu$m below the sample surface a weak bimodal distribution exists reflecting an alternation between about 5 $\upmu$m and 23 $\upmu$m spacing. With increasing depth a unimodal distribution with a nearly unchanged average spacing of 13.6 $\upmu$m is quickly approached, and at 19 $\upmu$m below the sample surface the crack spacings show close to Gaussian normal distribution about the mean of 13.9 $\upmu$m. The initial change in the distribution of the crack spacings results from systematic turning and, in places, oscillating of the individual cracks in the first few $\upmu$m below the sample surface. Further into the sample, the mean crack spacing increases successively. For depths of $> 60$ $\upmu$m below the sample surface the increase in average crack spacing becomes more pronounced, and the crack spacings become more widely distributed. Both features reflect the successive decrease in overall crack number with increasing depth. This is due to the fact that some cracks stopped growing at smaller depth (Predan et al. 2020).

**Fig. 4:**
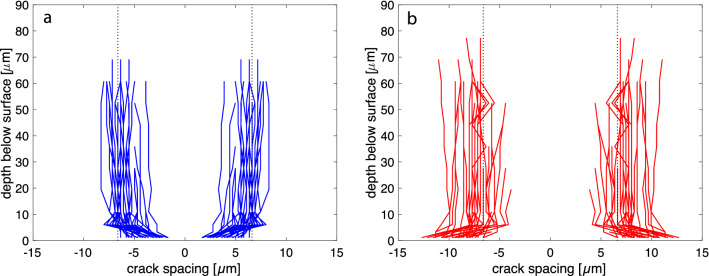
Spacing between cracks evolved from pairs of cracks with lower than average spacing (**a**) and with larger than average spacing (**b**) on the sample surface; the vertical dotted lines indicate average crack spacing; the data are from the 7 day experiment

In Fig. 4a, the evolution of the crack spacing with depth is shown for pairs of cracks with particularly close spacing on the sample surface. The pairs were selected based on the criterion that, on the sample surface, their spacing is below the average crack spacing and the spacing to the neighboring cracks on both sides of the pair is above the average crack spacing. The opposite criteria were applied for selecting pairs with particularly wide spacing on the sample surface (Fig. 4b). For the pairs of initially closely spaced cracks the spacing increases and for the pairs of initially widely spaced cracks the spacing decreases over the first few micrometers beneath the sample surface. In both cases, the average crack spacing is quickly approached.

**Fig. 5:**
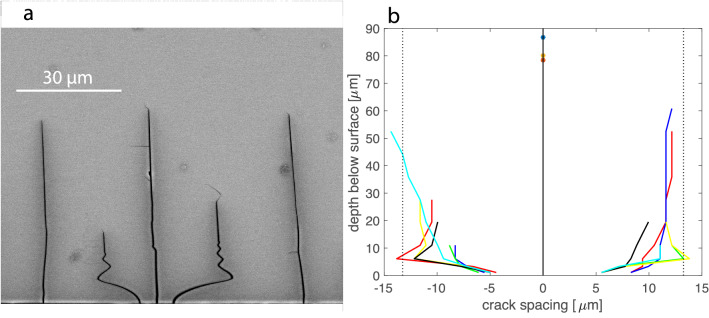
**a** High contrast BSE image of crack triple with a nearly straight central crack and two flanking cracks with typical wavy propagation paths; **b** spacing between cracks evolved from triples of closely spaced crack nuclei; the vertical dotted lines indicate the average crack spacing: The potentially occurring oscillations of the crack path such as seen in (**a**) are not resolved in (**b**), because this diagram is based on the measurements of distances between neighboring cracks, which were only done at certain depths below the sample surface (see Fig. 3); the data are from the 7 day experiment

In Fig. 5, the evolution of the crack spacing with depth below the sample surface is shown for triples of initially closely spaced cracks. The triples were selected based on the criterion that three neighboring cracks are separated by distances that are below the average spacing on the sample surface and the neighboring cracks on both sides of the triple occur at distances above the average spacing. Taking the central crack as reference, it is seen that the two cracks flanking the central crack on both sides quickly diverge from the central crack and assume a position that closely approaches the average crack spacing. In several cases, the flanking cracks diverge from the central crack so strongly that the distance from the central crack exceeds the average crack spacing. These cracks turn back towards the central crack and finally propagate into depth at a position that closely corresponds to the average crack spacing. Some of these cracks show multiple turning and oscillation between the central and the neighboring crack (Fig. 5a). We call them *wavy cracks*.

**Fig. 6:**
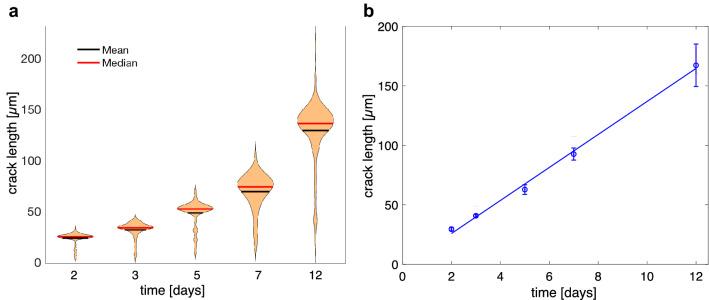
**a** Violine plots showing the distribution of crack length after 2 days, 3 days, 5 days, 7 days, and 12 days of Na–K exchange; **b** average length versus time for the cracks the length of which fall into the upper 10% of the crack length produced after a specific run duration

The distribution of crack length is shown for different run durations in Fig. 6a. Overall, the crack length increase with increasing run duration. The distribution of crack length is asymmetrical about the mean with a relatively narrow distribution towards values above the mean length and a considerably more extended distribution to values below the mean length. For run durations of $\ge 3$ days, distinct sub-maxima appear at short crack length, reflecting the fact that a fraction of the cracks stopped at a relatively early stage of the experiment. The average length of the cracks belonging to the longest 10% ones are plotted versus run duration in Fig. 6b. The crack length show a linear increase with run duration reflecting a constant growth rate of $1.6 \times 10^{-10}$ m/s.

### Compositional patterns associated with the cracks

**Fig. 7:**
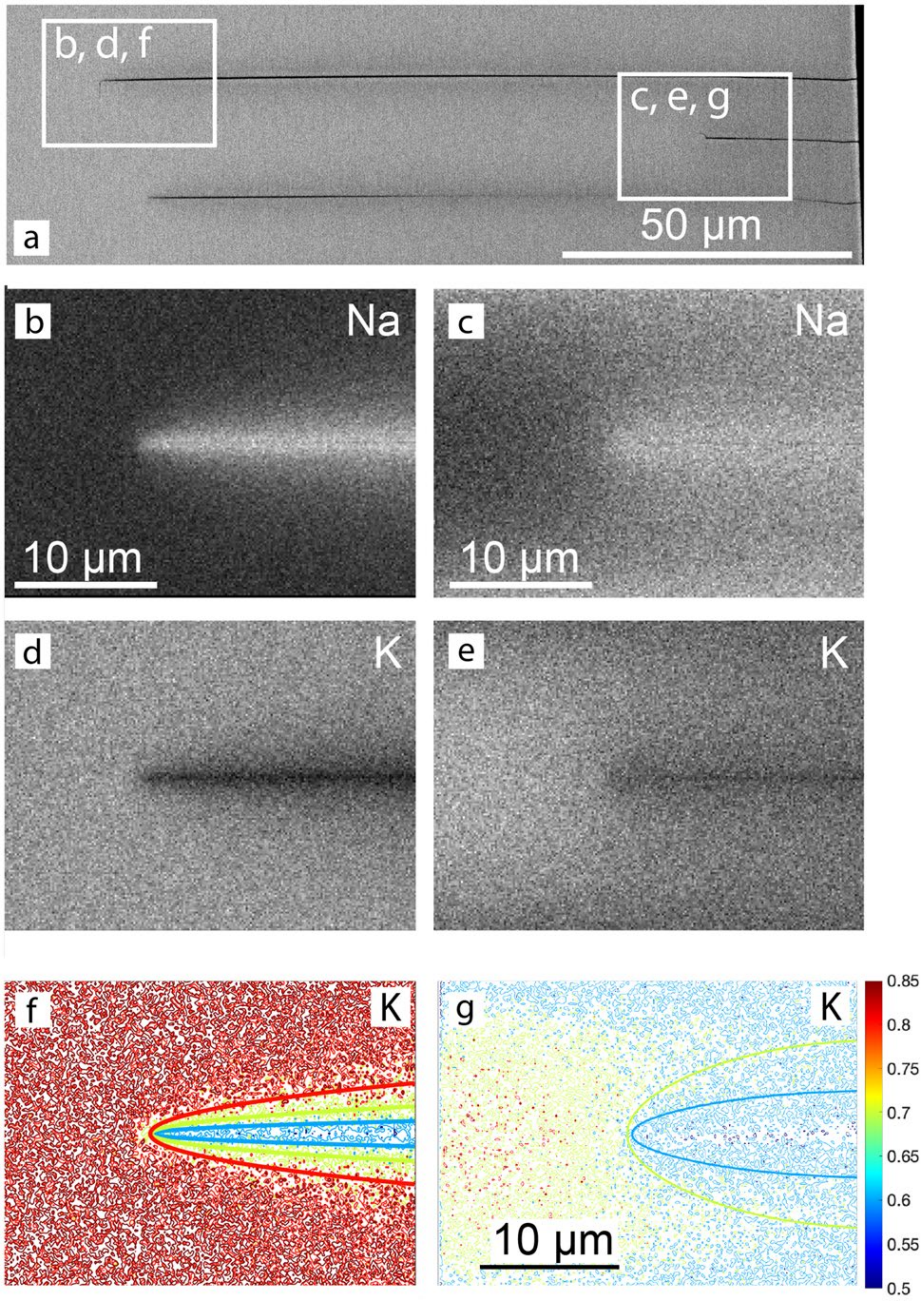
**a** BSE overview image, **b**, **c** Na-distribution maps of long (left) and short (right) crack, d), **e** K-distribution maps of long (left) and short (right) crack, **f**, **g** contour maps for K-mole fraction; the irregularly shaped thin lines are the contours obtained from quantitative element maps, the heavy solid lines are calculated from a 2D diffusion model; the data are from the 12 day experiment

BSE images as well as K and Na element distribution maps are shown for cracks from the 12 day experiment in Fig. 7. Na is enriched and K is depleted along the cracks relative to the background concentrations further away from the cracks. Quantitative point analyses revealed that the background composition corresponds to the original composition of the feldspar with $c_{\mathrm{K}} = 0.85$. At the crack flanks the composition approaches $c_{\mathrm{K}} = 0.50$, which is the equilibrium composition of alkali feldspar coexisting with an NaCl–KCl melt with $c_{\mathrm{KCl}} = 0.25$ at 850$^\circ$C and ambient pressure. The Na-enriched and K-depleted zone around the crack tip has the shape of an acute arrow head for the long crack (left panels), and it has the shape of a comparatively dull arrow head for the short crack (right panels). Moreover, for the long crack the background composition is reached at a distance of $\ge$3 $\upmu$m in front and aside of the crack tip (Fig. 7f). In contrast, for the short crack the Na content is elevated above the background composition everywhere between the short crack and the neighboring long cracks (see Fig. 7g).

## Discussion

### Crack growth rate

Ultimately, the formation and propagation of the parallel cracks are driven by the exchange of Na and K between the original alkali feldspar and the NaCl–KCl salt melt. At the experimental conditions, the original feldspar is not in equilibrium with the salt melt and is shifted towards the equilibrium composition by cation exchange. In the initial phase of the experiment, a Na-rich surface layer forms by the diffusion mediated cation exchange between the feldspar and the salt melt, and a tensile stress oriented parallel to the sample surface develops in the chemically altered layer. Once a critical tensile stress of about 300 MPa (Neusser et al. 2012) is exceeded, fracturing occurs, and mode I edge cracks form approximately perpendicular to the crystallographic ${\mathbf {a}}$ direction. This happens quite early in the experiment. The flanks of the newly formed cracks serve as additional surfaces for Na–K cation exchange, and a halo of more Na-rich feldspar develops around each crack. The associated lattice contraction and the resulting tensile stresses drive further crack growth. Crack propagation at constant velocity is due to the intimate coupling between diffusion-mediated cation exchange and fracturing, which leads to a quasi stationary state situation with respect to a coordinate system moving with the crack tip (Petrishcheva et al. 2019). In our experiments, crack growth with constant velocity is well documented for the cracks pertaining to the longest 10% cracks at a given run duration (Fig. 6b). This suggests that the propagation of the longest cracks is indeed exclusively controlled by the stress state caused by the diffusion-mediated evolution of the concentration and the according eigenstrain fields around the crack tips.

Due to mechanical interaction between neighboring crack tips, the growth of initially equally long parallel edge cracks may undergo a certain *growth instability* leading to a bifurcation in the rate of crack propagation. Some cracks stop, while other ones continue growing (Bazant and Ohtsubo 1977; Bazant et al. 1979; Sumi et al. 1980; Keer et al. 1979). Such a dichotomy in growth rate is evident from the observation of long and short cracks in our experiments (Figs. 2 and 7) and was analyzed in detail by Predan et al. (2020). Numerical simulations by Predan et al. (2020) indicate that the propagation velocity of the cracks decreases after each bifurcation event. In our experiments this effect was too subtle to be discerned. The contrasting crack propagation rates also manifest themselves in the shapes of the Na-enriched and K-depleted halos around the tips of the long, continuously growing, and the short, early stopped, cracks, which have the shapes of acute and dull arrow heads, respectively. In Figs. 7f, g, the irregularly shaped lines are concentration contours obtained from calibrated element distribution maps. The smooth contours were calculated from the diffusion model suggested by Petrishcheva et al. (2019) using the diffusion coefficients given in Table 1. In the numerical simulation, the crack growth rate was $1.6 \times 10^{-10}$m/s over the entire experimental run duration of 12 days for the long crack. For the short crack, the growth rate was $1.6 \times 10^{-10}$m/s for the first 2 days and then was reduced by a factor of 20 for the remaining 10 days. Good agreement between the calculated and measured concentration fields indicates that for the long crack, the compositional pattern developed at a propagating crack tip for the total experimental run duration. In contrast, the concentration field around the short crack developed around an essentially stagnant crack tip for most of the run duration. This corroborates the interpretation that the short crack essentially stopped growing well before the end of the experiment, while the long crack grew until the sample was quenched.

### Energetics of the process

The compositional change of the feldspar (and the salt melt) due to cation exchange leads to lowering the system Gibbs energy. This Gibbs energy change is available for dissipation in the course of the processes underlying system evolution including Na–K interdiffusion and fracturing. In addition, part of this energy is stored in the surface energy of the newly formed crack flanks.

Following the seminal work of Coleman and Gurtin (1967) we describe the evolving system in terms of internal variables. In the case at hand, the standard set of the diffusing species concentrations is expanded to include crack length *a*. The negative derivative of the Gibbs energy with respect to the crack length, $-\partial G/\partial a$, quantifies the thermodynamic force driving crack growth, and the rate *da*/*dt* defines the induced thermodynamic flux. In what follows, we refer to the *Thermodynamic Extremal Principle* as introduced by Ziegler (1983), who extended Onsager’s work Onsager (1931). The principle implies that an adiabatic system that is out of equilibrium evolves along a path that ensures maximum rate of entropy production (Hackl and Fischer 2008; Fischer et al. 2014). The entropy is at a maximum and entropy production vanishes, when an equilibrium state is reached. For isothermal conditions the principle implies system evolution ensuring *maximum dissipation rate* that is maximum rate of Gibbs energy decrease. Accordingly, in equilibrium, Gibbs energy is minimized, and dissipation vanishes. In a stationary system subject to fluxes the dissipation rate is at a minimum (De Groot and Mazur 1984). The experimentally observed crack propagation in alkali feldspar is transient, and its evolution is expected to be governed by maximum dissipation rate. Nikolaevskij (1987) and later Slepyan (1993) were among the first authors who applied the maximum dissipation rate for studying crack growth in elastic bodies. Dissipation-based paths are also followed in ductile fracture (Verhoosel et al. 2009; Wambacq et al. 2021). An according variational treatment of thermo-chemo-mechanical processes has been published recently (Romero et al. 2021). Insofar, the concept of maximum dissipation rate can be considered as an actual and efficient approach for studying crack growth also in configurations that are subtle with respect to the path itself and the material the crack propagates in.

In the case at hand, *diffusion* and *crack propagation* are the dissipative processes underlying system evolution. Both processes are intimately coupled via the compositional eigenstrain state and the associated stress state. In the following, we investigate to what extent the rate of free energy dissipation associated with the evolution of the system of cracks depends on their spatial distribution.

#### Simulation-cell analysis

**Fig. 8:**
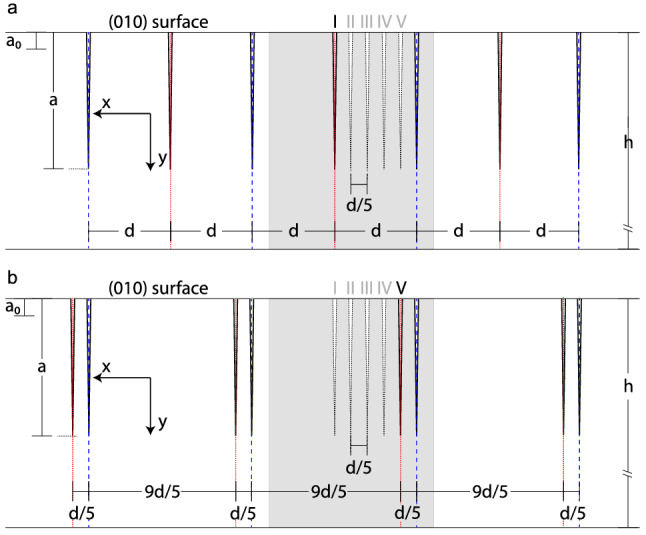
Schematic drawing of system geometry projected along the *z* direction: a periodic array of parallel cracks with crack spacing 2*d* (blue dashed lines) is considered with intermediate cracks (red dotted lines) placed at a fixed position between any two neighboring 2*d*-spaced cracks. The indices I–V mark the different configurations regarding the positions of the intermediate cracks; **a** configuration I, **b** configuration V; $a_0$ and *a* are the initial and the actual crack lengths, respectively. The grey shaded area is the simulation cell comprising two parallel cracks

To investigate how the dissipation rate develops as a function of the position of an intermediate crack between two neighbor cracks, we consider a system of parallel cracks in a (010) plate of alkali feldspar (Fig. 8). In this thought experiment the cracks emanate from the top (010) surface, and we assume that they propagate into the plate as straight cracks perpendicular to the (010) surface. The local orthogonal coordinate system *Oxyz* is generated by the vectors ${\mathbf {a}}$, ${\mathbf {b}}$, and ${\mathbf {c}}^\star \parallel {\mathbf {a}}\times {\mathbf {b}}$ (see Fig. 1). In this coordinate system the (010) cracks extend in the *y*–*z* plane, and the (010) surface of the plate is parallel to the *x*–*z* plane. The average crack spacing is *d*. Calculations are performed for a simulation cell covering $2d = 28$ $\upmu$m in *x*-direction, $h = 500$ $\upmu$m in *y*-direction and 1000 $\upmu$m in *z*-direction (grey shaded area in Fig. 8). The top surface is a free boundary, and periodic boundary conditions are applied at the left and right boundaries. In the initial configuration all cracks have the length $a_0$, which was chosen as $a_0 = 5$ $\upmu$m. Diffusion-mediated crack propagation is modeled using the method presented by Predan et al. (2020), and for computational details the reader is referred to this work. For the simulation the final crack length was set to ${\bar{a}} = 45$ $\upmu$m. The extension *h* of the simulation cell in *y* direction is taken as $h \ge 10\, {\bar{a}}$, to avoid any effects of limited system size. A 2D generalized plain strain model is assumed, thus in *z*-direction the cracks extend throughout the simulation cell.

Five different scenarios are considered with respect to the distribution of the cracks. For all configurations, the spacing between every second crack is 2*d*. In configuration I, the intervening *intermediate* crack is positioned in the middle between the two neighboring 2*d* spaced cracks so that an array of equally spaced parallel cracks with crack spacing *d* is obtained (Fig. 8a). For all other configurations, the intermediate crack is at an eccentric position relative to the neighboring 2*d* spaced cracks. In configuration V, which is the most extreme case of non uniform crack distribution, the intermediate crack is positioned *d*/5 from the right and 9*d*/5 from the left neighbor crack (Fig. 8b). This produces an array of pairs of *d*/5 spaced cracks that are separated by a distance of 9*d*/5. Configurations II, III and IV are intermediate cases forming a transition from configuration I (uniform spacing) to configuration V (most pronounced non-uniform spacing).

We investigate the total dissipation rate associated with the evolution of parallel cracks according to configurations I–V. This procedure allows to identify the most probable configuration that a system of chemically induced cracks in an anisotropic material will choose. According to the Thermodynamic Extremal Principle this configuration should be the one for wich the dissipation rate is the highest.

#### Dissipation rate due to diffusion

Regarding the dissipation due to diffusion it must be noted that Na and K in the alkali sub-lattice of alkali feldspar are rather mobile, whereas Al and Si in the 3D framework of AlO$_4^{5-}$ and SiO$_4^{4-}$ tetrahedra are tightly bound to oxygen. As a consequence, neither oxygen nor Al or Si diffuse to any appreciable extent during Na–K cation exchange (Neusser et al. 2012; Petrishcheva et al. 2014; Schaeffer et al. 2014; Petrishcheva et al. 2020). Thus only the interdiffusion of Na and K in the alkali sub-lattice contributes to the dissipation of free energy. Vacancies are neither generated nor annihilated and, due to charge balance constraints, no flux of vacancies occurs. In the gem quality alkali feldspar used, the concentration of vacancies is low allowing to assume $c_{\mathrm{Na}} + c_{\mathrm{K}} =1$ with $c_{\mathrm{Na}}$ and $c_{\mathrm{K}}$ as the Na and K site fractions on the alkali sub-lattice; we set $c_{\mathrm{K}} \equiv c$. This implies that the diffusive fluxes of Na and K are coupled so that ${\mathbf {j}}_{\mathrm{Na}} + {\mathbf {j}}_{\mathrm{K}} = {\mathbf {0}}$.

The dissipation rate due to diffusion in a given volume $\Omega$ reads

1 $$\begin{aligned} Q_{\mathrm{diff}} = \int _{\Omega } q_{\mathrm{diff}} \mathrm{d}\Omega = \int _{\Omega } \left( \sum _{i} j_i X_{i} \right) \mathrm{d}\Omega , \quad j_i = \sum _{j} L_{ij} X_j, \end{aligned}$$

where $q_{\mathrm{diff}}$ is the dissipation rate density, and $j_i$ are the diffusive fluxes originating from the thermodynamic forces $X_i$. ${\mathbf {L}}_{ij}$ represents the mobility coefficients that are related to the so-called Onsager coefficients. In the case at hand, only one species diffuses independently, and the indices $i, j$ in Eq. 1 label space coordinates. From Eq. 1 one obtains

2 $$\begin{aligned} Q_{\mathrm{diff}} = \int _{\Omega } \left( \sum _{ij} {\tilde{L}}_{ij} \, j_{i} \, j_{j} \right) \mathrm{d}\Omega , \quad \tilde{{\mathbf {L}}} = {\mathbf {L}}^{-1}. \end{aligned}$$

We investigate cracks parallel to the $y$-$z$ plane and assume that the concentrations only depend on the $x$ and $y$ coordinates and on time. In this case, the general mobility matrix for a monoclinic feldspar reduces to a diagonal 2$\times$2 matrix. The dissipation rate due to the interdiffusion of Na and K then reads (Fischer and Svoboda 2014)

3 $$\begin{aligned} Q_{\mathrm{diff}} = \int _{\Omega } \left( \frac{ j_x^2}{L_{xx}} + \frac{ j_y^2}{L_{yy}} \right) \mathrm{d}\Omega . \end{aligned}$$

For the mobility coefficients $L_{xx}$ and $L_{yy}$, we have (Petrishcheva and Abart 2012)

4 $$\begin{aligned} L_{xx} = \frac{D_{xx}}{\bar{\Omega }R_\mathrm{g} T} c (1-c), \quad L_{yy} = \frac{D_{yy}}{\bar{\Omega }R_\mathrm{g} T} c (1-c). \end{aligned}$$

The quantities $D_{xx}$ and $D_{yy}$ are the Na–K interdiffusion coefficients in $x$ and $y$ direction, $\bar{\Omega }$ is the average molar volume of the alkali feldspar solid-solution, $R_\mathrm{g}$ is the gas constant, and $T$ is absolute temperature.

The two-dimensional diffusion equation for the K site fraction $c=c(x,y,t)$ reads

5 $$\begin{aligned} \partial _t c = D_{xx} \partial _x^2 c+ D_{yy} \partial _y^2 c, \end{aligned}$$

and is solved for the simulation domain numerically. Constant concentration boundary conditions, corresponding to constant K site fraction, are applied at the sample surface and at the crack flanks, which are all assumed to be in equilibrium with the salt melt. Periodic boundary conditions are applied at the side walls of the simulation cell. A zero flux boundary condition is applied at the far end boundary of the simulation cell, where diffusive fluxes are negligible throughout the entire simulation period. The Na–K interdiffusion coefficients for the conditions of the experiment are given in Table 1. In the compositional range of interest ($0.50 \le c \le 0.85$) the compositional dependence of the diffusivities is minute (Schaeffer et al. 2014; Petrishcheva et al. 2020), consequently, these values may be regarded as constants.

The parameters used for calculating the dissipation rate due to diffusion are given in Table 1. The molar volume of alkali feldspar with composition $c$ = 0.67, which is intermediate between the original and the equilibrium compositions, is calculated from $\bar{\Omega }_{\mathrm{sanidine}} = 1.108 \times 10^{-4}$ m$^3$/mol and $\bar{\Omega }_{\mathrm{high-albite}} = 1.034 \times 10^{-4}$ m$^3$/mol applying Vegard’s rule. At 1 bar and 850$^{\circ }$C, $\bar{\Omega }$ is equal to $1.084 \times 10^{-4}$ m$^3$/mol. Accordingly, we obtain

$$\begin{aligned} L_{xx} = 4.74 \times 10^{-17} c (1-c) \quad \mathrm{mol^2/\left( sNm^2\right) } \\ L_{yy} = 5.34 \times 10^{-18} c (1-c) \quad \mathrm{mol^2/\left( sNm^2\right) }, \end{aligned}$$

for the mobility coefficients. The mobility coefficients could also be derived from tracer diffusion coefficients, which is perfectly equivalent to using interdiffusion coefficients, when Mannings equation (Manning 1968) for binary interdiffusion (his Eqs. 1–27) is substituted for the interdiffusion coefficient. We use the interdiffusion coefficient, because this was experimentally determined including compositional and direction dependence for the same felspar as used in the fracturing experiments (Schaeffer et al. 2014), whereas Na and K tracer diffusion coefficients are only available for the direction perpendicular to (001) (Petrishcheva et al. 2020).

**Table 1:** System parameters used for calculating $Q_{\mathrm{diff}}$ and $Q_{\mathrm{mech}}$

Parameter	Symbol	Numerical value	Units
Temperature	*T*	850/1123	$^\circ$C/K
Molar volume of feldspar	$\bar{\Omega }$	$1.084 \times 10^{-4}$	m$^3$mol$^{-1}$
Na/K interdiff. coefficient $\parallel x$ $^\mathrm{a}$	$D_{xx}$	$4.8\times 10^{-17}$	m$^2$s$^{-1}$
Na/K interdiff. coefficient $\parallel y$ $^\mathrm{a}$	$D_{yy}$	$5.4\times 10^{-18}$	m$^2$s$^{-1}$
Crack growth rate	${\dot{a}}$	$1.6 \times 10^{-10}$	m s$^{-1}$
Critical value of the J-integral	$J_c$	2.0	N m$^{-1}$
Half width of simulation cell	*d*	$1.4\times 10^{-5}$	m
Thickness of simulation cell $\parallel z$	${\bar{z}}$	$1 \times 10^{-3}$	m
Initial crack length	$a_0$	$5\times 10^{-6}$	m
Final crack length	${\bar{a}}$	$45\times 10^{-6}$	m

$^\mathrm{a}$Taken from Petrishcheva et al. (2014)

#### Dissipation rate due to fracturing

The dissipation rate due to fracturing is related to the irreversible energy provision associated with formation of new crack surfaces. The dissipation rate per unit thickness ($z$ direction in the case at hand) due to propagation of one crack reads

6 $$\begin{aligned} Q_{\mathrm{mech}} = -\frac{\mathrm{d} \Pi }{\mathrm{d} a} {\dot{a}}, \end{aligned}$$

where $- \mathrm{d} \Pi / \mathrm{d}a$ is the change in the total potential energy of an elastic system with a change in the crack length $a$, and ${\dot{a}}$ is the rate of crack growth. Using the definition of the so-called *J*-integral (Cherepanov 1967; Rice 1968)

7 $$\begin{aligned} \textit{J}=-\frac{\mathrm{d} \Pi }{\mathrm{d} a}, \end{aligned}$$

the contribution to the dissipation by fracturing reads (Kolednik et al. 2010)

8 $$\begin{aligned} Q_{\mathrm{mech}} = J_\mathrm{c} {\dot{a}}, \end{aligned}$$

where $J_\mathrm{c}$ is the critical value of the *J*-integral, that is the minimum value required for a crack to propagate. We refer to Slepyan (1993), who pointed out that some fraction of the *released* energy is stored as the surface energy associated with the newly formed crack flanks and is thus not dissipated. The critical value $J_\mathrm{c}$ in Eq. 8 is, however, only related to the dissipation process. The calculation of the J-integral is described in the supplementary information, and the respective system parameters are given in Table 1.

### Crack spacing and overall dissipation rate

Regarding $Q_{\mathrm{mech}}$, we assume a critical energy release rate of $J_\mathrm{c}$ = 2.0 Jm$^{-2}$, which was determined experimentally earlier (Petrishcheva et al. 2019). The corresponding rate of surface energy associated with the newly formed crack flanks is 2$\gamma _\mathrm{s} {\bar{h}} {\dot{a}}$, where $\gamma _\mathrm{s}$ is the surface energy per unit area, and ${\bar{h}}$ is the extension of the system in *z* direction. For any reasonable feldspar surface energy this amounts to $\le 0.3$% of the free energy change due to cation exchange of the feldspar and may be neglected (see supplementary information). With the above value of $J_\mathrm{c}$ and using the simulation cell described earlier, the simulations yield a crack growth rate, which correspond well with the experimentally observed one. Inserting these values for $_\mathrm{c}$ and ${\dot{a}}$ into Eq. 8, the dissipation rate due to propagation of the two cracks in the simulation cell is calculated as $Q_{\mathrm{mech}} = 6 \times 10^{-13}$Js$^{-1}$ for the instant, when $a = 45$ $\upmu$m.

**Fig. 9:**
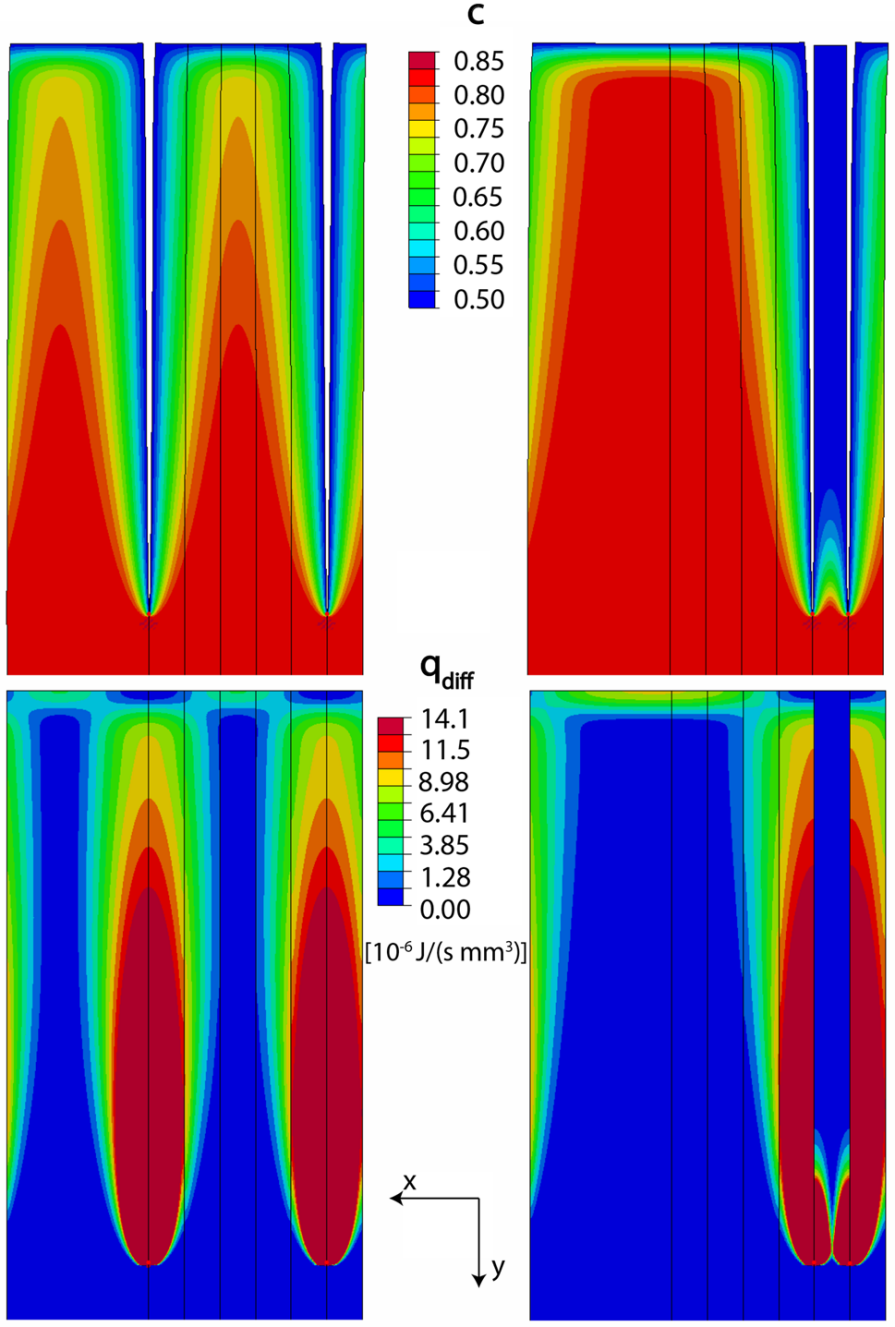
Spatial distribution of composition *c* and dissipation rate density due to diffusion $q_{\mathrm{diff}}$ calculated for configurations I and V at an instant, where the cracks have grown to a length of $45 \upmu$m.

Regarding $Q_{\mathrm{diff}}$, we refer to Fig. 9, where the fields of feldspar composition and of the local dissipation rate due to diffusion are shown. It is seen that each crack is accompanied by an oval shaped field of elevated dissipation rates. For configuration I, the fields associated with the two cracks in the simulation cell are largely separated from each other. In contrast, for the two closely spaced cracks in configuration V, the fields associated with the two cracks overlap, and a domain with low dissipation rate, which corresponds to an area with low diffusive flux, is generated between the two cracks. As a consequence, the dissipation rate due to diffusion is lower for configuration V than for configuration I.

Based on the calculated diffusive fluxes and using Eq. 3 with $\Omega$ being the volume of the simulation cell, the rate of free energy dissipation due to diffusion is $Q_{\mathrm{diff}} (\mathrm{I}) = 1.84 \times 10^{-10}$ Js$^{-1}$, and $Q_{\mathrm{diff}} (\mathrm{V}) = 1.23 \times 10^{-10}$ Js$^{-1}$ for configurations I and V, respectively, in both cases $a = 45$ $\upmu$m.

Recalling that $Q_{\mathrm{mech}} = 6 \times 10^{-13}$Js$^{-1}$, it emerges that for the considered configurations $Q_{\mathrm{diff}}$ is by a factor of about 400–600, depending on configuration, larger than $Q_{\mathrm{mech}}$. Noting that the diffusive flux at all surfaces, including the diffusive flux at the flanks of the already developed cracks, enters the integrand in Eq. 3, while $Q_\mathrm{mech}$ only reflects the dissipation due to the incremental generation of new surfaces at the crack tips, this is not surprising. Hence, diffusion provides the by far dominant contribution to the overall dissipation rate.

Using thermodynamic data from Holland and Powell (2011) the molar Gibbs energy of the exchange reaction is calculated as $\Delta _\mathrm{r} g = -5015$ J/mol at the conditions of the experiment (see supplementary information). This corresponds to a total Gibbs energy change of $-6.5 \times 10^{-4}$ J for the simulation cell (see supplementary information). Given the crack growth rate of ${\dot{a}} = 1.6 \times 10^{-10}$ m/s, it would take $3.12 \times 10^{6}$s for the crack to grow to a length of 500 $\upmu$m, the length of the simulation cell in *y* direction. A full equilibration of the feldspar contained in the simulation cell within this time would correspond to an average free energy rate of ${\dot{G}}=-2.1 \times 10^{-10}$ J/s. This is well in line with the calculated dissipation rates. The calculated rate of free energy change is somewhat higher than the calculated dissipation rates, because its calculation is based on the assumption of complete equilibration of the feldspar in the simulation cell with the salt melt. For the cracks to propagate, however, only partial equilibration of the feldspar is required. In addition, some of the energy is stored in the surface energy of the newly formed crack flanks. It is generally found that fracturing proceeds at thermodynamic driving forces that are larger than those needed to create the new crack surfaces. The surplus may fuel additional dissipative processes (Cramer et al. 2000).

**Fig. 10:**
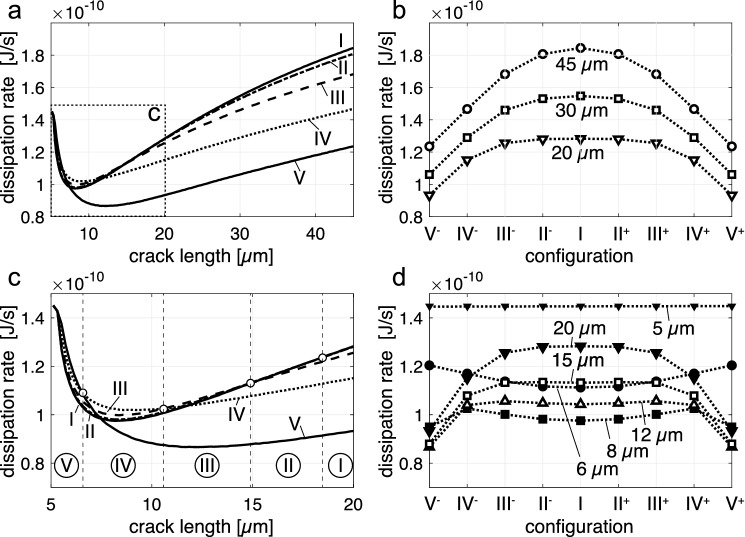
**a** Evolution of the dissipation rate for each of the different configurations I–V with increasing crack length. The insert labelled “c” in diagram (**a**) corresponds to the close-up diagram in (**c**). In (**b**, **d**), the dissipation rates for configurations I–V are compared for different crack length. Configurations II–V are drawn for both, left (“$^-$”) and right (“$^+$”) of the central position. **c** Close up of diagram for the early evolutionary stages, the roman numerals in circles mark the configuration with the highest dissipation rate in the respective range of crack length

The evolution of the dissipation rate for the different configurations I–V is shown in Fig. 10. In Fig. 10a, c it is seen that the total dissipation rate starts at the same value for all crack configurations and shows a pronounced decrease during the incipient stages of crack growth. This is due to the fact that diffusion from the specimen surface and from the flanks of the pre-existing cracks of length $a_0$ is similarly efficient for all crack configurations, as long as the diffusion fields of neighboring cracks are separated from each other, which is the case for the early stages of the process. The initial decrease of the total dissipation rate for all configurations arises from the fact that, given the constant concentration boundary conditions at the sample surface and at the crack flanks, diffusion becomes successively less efficient as the compositional gradients decrease with time. The pre-existing cracks of length $a_0$ are stagnant initially and only start to grow, when the chemically induced stresses at the crack tips become sufficiently large. Only, when new surfaces for cation exchange are generated by crack growth, the dissipation rate goes through a minimum and then increases with growing crack length.

At a crack length of about 7 $\upmu$m, the total dissipation rate begins to diverge for the different configurations. During this stage, the diffusion fields accompanying the cracks start to overlap to different degrees for the different configurations. During incipient crack growth, the dissipation rate is maximized for configuration V (Fig. 10c, e). With increasing crack length in the range of 6–20 $\upmu$m the configuration with highest dissipation rate changes following the sequences V–IV–III–II–I. At a crack length of more than about 18 $\upmu$m configuration I (uniform crack spacing) is the one with the highest dissipation rate (Fig. 10a, b). At these crack lengths, the individual values of the dissipation rate decrease monotonically from configuration I to configuration V. This occurs, because the degree of overlap between the diffusion fields of neighboring cracks increases from uniformly spaced cracks to the configurations with successively more non-uniform crack spacing. Thus, after the initial phase of fracturing, configuration I becomes the one with the highest dissipation rate and thus the most probable one according to the Thermodynamic Extremal Principle. Actually configuration I, uniform crack spacing, is experimentally observed in the fully developed crack patterns.

**Fig. 11:**
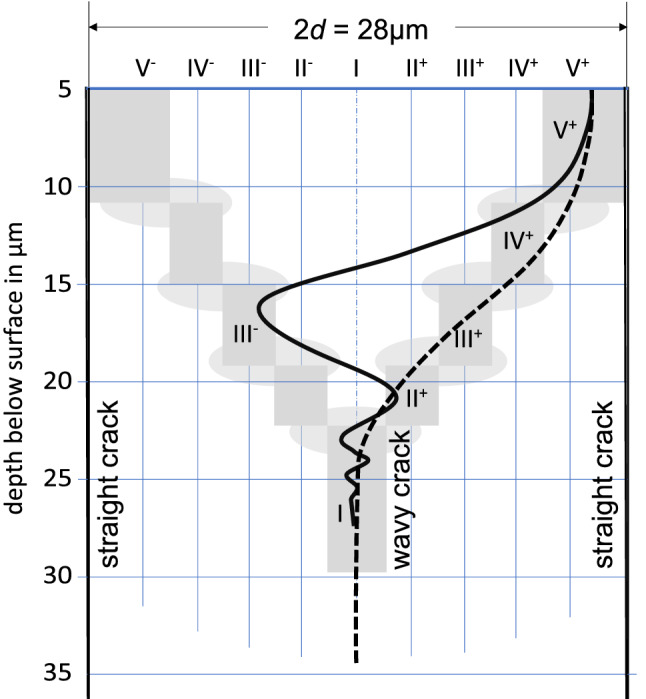
Exemplary propagation path of wavy crack (solid curved line) and simply turning crack (dashed line) that nucleated close to the right neighbor crack (solid vertical line)

The thermodynamic extremal principle also serves as a rationale for the early evolution of the cracks including their systematic turning and oscillations. In Fig. 10d the dissipation rate of the five different configurations is shown for crack length of 6 $\upmu$m, 8 $\upmu$m, 12 $\upmu$m, 15 $\upmu$m, and 20 $\upmu$m. It is seen that with increasing crack length the position of the intermediate crack with maximum dissipation rate successively shifts from an eccentric towards a central position between the two neighboring 2*d* spaced cracks. Actually, such a path is frequently observed for cracks that nucleated eccentrically between two neighboring cracks (Figs. 4 and 5). It should be noted that for crack length below 20 $\upmu$m two positions of the intermediate crack exist, where the rate of free energy dissipation is maximized, and that these positions are distributed symmetrically about the central position (Fig. 10d). Thus, a crack propagating in an eccentric position on one side of the central position may find an equally probable position on the other side of the central position. As a consequence, switching sides may occur, and multiple switching may give raise to an “oscillating” crack path. In contrast to the observation of stationary oscillations of a single crack in a moving temperature or electric field, as treated in Addabedia and Pomeau (1995), Deegan et al. (2003), Bouchbinder et al. (2003), Niefanger et al. (2004), Menouillard and Belytschko (2011), here the oscillations give the impression of being damped, a phenomenon that is indeed frequently observed in our experiment (Fig. 5a). This is because the position of maximum dissipation rate successively shifts towards more central positions. The oscillations observed in our experiments can thus be interpreted as a sequence of V$^+$–IV$^-$–III$^+$–II$^-$ I, where the superscripts “$^+$” and “$^-$” indicate right and left from the central position, respectively, and the amplitude of the oscillation decreases monotonically (Fig. 11).

In summary, the system of chemically induced edge cracks organizes itself into a configuration with approximately uniform crack spacing, although the initial crack spacing is markedly non-uniform. For each evolutionary stage the observed crack patterns correspond to configurations that maximise the dissipation rate. The criterion of maximum rate of free energy dissipation combined with classical crack theory has already been applied in the context of purely mechanically driven crack propagation by Slepyan (1993). At this point, we emphasize that for chemically driven crack propagation, the development of the global configuration is controlled almost exclusively by the diffusion process. The details of the individual crack propagation paths are, however, mainly controlled by the local stress state near the crack tip resulting from the eigenstrains generated by the diffusion-mediated compositional change of the material in the immediate vicinity of the crack. Consequently, the mechanical material properties also play an important role. A rigorous analysis of the local thermodynamic forces controlling the crack propagation paths is, however, beyond the scope of this communication. A qualitative assessment is given in the supplementary information.

Our considerations can be generalized to fracturing induced by heterogeneous eigenstrain state, irrespective of the process underlying volume and/or shape change. Several studies addressing such problems can be found in the literature. For example, self fracturing caused by the shrinkage due to volatile loss during thermal decomposition of a solid was considered by Yakobson (1991). The basic problem is similar to what is addressed in our study, but in detail his analysis is different from our approach. Yakobson (1991) considered a crack-network and derived expressions for the rate of crack-network propagation and for the diameter of the resulting blocks. In contrast to our model, an isotropic solid was assumed, and interaction between cracks as well as concentration fields around individual cracks were disregarded. Malthe-Sorenssen et al. (2006) extended this model to account for diffusion mediated changes in the eigenstrain state around individual cracks and provided numerical simulations of fracturing in a chemically decomposing solid using discrete element modelling. These models produce highly realistic crack networks (Ulven et al. 2017) and have been applied successfully for simulating hydraulic fracturing Ulven et al. (2014a, 2014b) and fracturing induced by swelling particles Kuleci et al. (2017). In general, discrete element models do, however, not account for the intrinsic anisotropy of mineral phases with low symmetry such as feldspar.

Another related problem is encountered in the context of thermally induced fracturing. A solid subject to directional cooling typically shrinks at the cold end, and extension cracks may be induced. Boeck et al. (1999) analysed the self propagation of crack arrays in a thermally loaded two-dimensional half-space. The problem is quite similar to the chemically driven fracturing in alkali feldspar, but these latter authors considered stationary propagation of equally spaced straight cracks in an isotropic medium. They derived expressions for crack spacing and propagation rate from a stability analysis of the stationary solution. Furthermore, wavy cracks have been described from a glass strip pulled from a hot oven into a cold bath Addabedia and Pomeau (1995), Bouchbinder et al. (2003), Menouillard and Belytschko (2011) from thermally loaded single crystals of silicon (Deegan et al. 2003) and from ferroelectric ceramics exposed to a moving electric field (Niefanger et al. 2004). Addabedia and Pomeau (1995) and Bouchbinder et al. (2003) performed stability analyses and Menouillard and Belytschko (2011) employed local symmetry criteria and numerical simulation for analysing the path of a single oscillating crack, where temperature was treated as a background field unrelated to the crack.

To the best of the knowledge of the authors, non of the above mentioned studies addressed the evolution from unevenly distributed towards evenly spaced cracks during crack propagation driven by the diffusion-mediated eigenstrain state around each individual crack. We consider the Thermodynamic Extremal Principle as a sound basis for rationalizing the experimental observations.

## Conclusions

The evolution of chemically induced edge cracks in alkali feldspar was investigated experimentally and discussed in the light of the Thermodynamic Extremal Principle. To this end, a chemical shift towards more Na-rich compositions was applied to cuboid plates of gem-quality K-rich alkali feldspar with polished (010) surfaces by diffusion-mediated cation exchange with an NaCl–KCl salt melt at 850 $^\circ$C and close to ambient pressure. The composition of the salt melt was chosen so that a Na-rich layer formed at and beneath the specimen surface. The associated anisotropic lattice contraction produced a tensile stress state in the chemically altered layer of the feldspar. When the largest tensile stress component, which is sub-parallel to the crystallographic $\mathbf{a}$-axis of feldspar, exceeded a critical value, fracturing approximately perpendicular to the crystallographic ${\mathbf {a}}$-axis occurred. The newly formed crack flanks served as additional surfaces for cation exchange, and a diffusion halo with elevated Na-contents developed around each crack. The associated lattice contraction and tensile stress state lead to further crack propagation with constant velocity. The cracks nucleated with non uniform spacing on the sample surface and, due to systematic turning along their early propagation paths, quickly attained a more uniform spacing. In some cases, multiple turning lead to peculiar wavy crack paths. This behavior ensures that the rate of free energy dissipation is maximized in every stage of the system evolution. The total dissipation rate associated with chemically driven fracturing in alkali feldspar comprises the dissipation rate due to crack growth and the dissipation rate due to Na–K interdiffusion, where the contribution due to diffusion dominates by far. The overall dissipation rate is independent of crack spacing during the incipient stages of crack growth. During later stages, when the diffusion halos accompanying the individual cracks start to overlap, the total dissipation rate is maximized when the crack spacing is uniform. In this configuration the overlap between the diffusion halos accompanying the individual cracks is minimized. Maximization of the overall dissipation rate is also ensured during the transitional stage, when the cracks develop from non uniform spacing on the specimen surface to nearly uniform spacing by systematic turning along their early propagation paths. The observed evolution of chemically induced cracks in gem quality alkali feldspar and its analysis provide a model for fracturing due to chemically induced and diffusion mediated negative eigenstrains in homogeneous anisotropic materials.

## Supplementary Information

Below is the link to the electronic supplementary material.

Supplementary file1 (DOCX 21136 kb)

## Data Availability

Not applicable.

## References

[CR1] Addabedia M, Pomeau Y (1995). Crack instabilities of a heated glass strip. Phys Rev E.

[CR2] Aditya S, Tapas NK, Samir PK (2017). Pre-treatment of rocks prior to comminution—a critical review of present practices. Int J Min Sci Technol.

[CR3] Angel R, Sochalski-Kolbus L, Tribaudino M (2012). Tilts and tetrahedra: the origin of the anisotropy of feldspars. Am Miner.

[CR4] Bazant Z, Ohtsubo H (1977). Stability conditions for propagation of a system of cracks in a brittle solid. Mech Res Commun.

[CR5] Bazant Z, Ohtsubo H, Aoh K (1979). Stability and post-critical growth of a system of cooling or shrinkage cracks. Int J Fract.

[CR6] Boeck T, Bahr H, Lampenscherf S (1999). Self-driven propagation of crack arrays: a stationary two-dimensional model. Phys Rev E.

[CR7] Bouchbinder E, Hentschel H, Procaccia I (2003). Dynamical instabilities of quasistatic crack propagation under thermal stress. Phys Rev E.

[CR8] Broz M, Cook R, Whitney D (2006). Microhardness, toughness, and modulus of Mohs scale minerals3. Am Miner.

[CR9] Cherepanov G (1967). Crack propagation in continuous media. J Appl Math Mech-USSR.

[CR10] Coleman B, Gurtin M (1967). Thermodynamics with internal state variables. J Chem Phys.

[CR11] Cramer T, Wanner A, Gumbsch P (2000). Energy dissipation and path instabilities in dynamic fracture of silicon single crystals. Phys Rev Lett.

[CR12] De Groot S, Mazur P (1984). Non-equilibrium thermodynamics.

[CR13] Deegan R, Chheda S, Patel L (2003). Wavy and rough cracks in silicon. Phys Rev E.

[CR14] Fischer FD, Svoboda J (2014). Diffusion of elements and vacancies in multi-component systems. Prog Mater Sci.

[CR15] Fischer FD, Svoboda J, Petryk H (2014). Thermodynamic extremal principles for irreversible processes in materials science. Acta Mater.

[CR16] Hackl K, Fischer FD (2008). On the relation between the principle of maximum dissipation and inelastic evolution given by dissipation potentials. Proc R Soc A-Math Phys Eng Sci.

[CR17] Holland TJB, Powell R (2011). An improved and extended internally consistent thermodynamic dataset for phases of petrological interest, involving a new equation of state for solids. J Metamorph Geol.

[CR18] Katsabanis P (2020). Analysis of the effects of blasting on comminution using experimental results and numerical modelling. Rock Mech Rock Eng.

[CR19] Keer L, Nemat-Nasser S, Oranratnachai A (1979). Unstable growth of thermally induced interacting cracks in brittle solids–further results. Int J Solids Struct.

[CR20] Kiselev A, Bachmann F, Pedevilla P (2017). Active sites in heterogeneous ice nucleation-the example of K-rich feldspars. Science.

[CR21] Kiselev A, Keinert A, Gaedeke T (2021). Effect of chemically induced fracturing on the ice nucleation activity of alkali feldspar. Atmos Chem Phys.

[CR22] Kolednik O, Predan J, Fischer FD (2010). Reprint of “Cracks in inhomogeneous materials Comprehensive assessment using the configurational forces concept”. Eng Fract Mech.

[CR23] Kroll H, Schmiemann I, von Coelln G (1986). Alkali feldspar solid-solutions. Am Miner.

[CR24] Kuleci H, Ulven OI, Rybacki E (2017). Reaction-induced fracturing in a hot pressed calcite-periclase aggregate. J Struct Geol.

[CR25] Malthe-Sorenssen A, Jamtveit B, Meakin P (2006). Fracture patterns generated by diffusion controlled volume changing reactions. Phys Rev Lett.

[CR26] Manning J (1968). Diffusion kinetics for atoms in crystals.

[CR27] Menouillard T, Belytschko T (2011). Analysis and computations of oscillating crack propagation in a heated strip. Int J Fract.

[CR28] Neusser G, Abart R, Fischer FD (2012). Experimental Na/K exchange between alkali feldspar and an NaCl-KCl salt melt: chemically induced fracturing and element partitioning. Contrib Miner Petrol.

[CR29] Niefanger R, Pham V, Schneider G (2004). Quasi-static straight and oscillatory crack propagation in ferroelectric ceramics due to moving electric field: experiments and theory. Acta Mater.

[CR30] Nikolaevskij V (1987). Path-independent rate integrals and the criterion of steady crack-growth in inelastic bodies. Eng Fract Mech.

[CR31] Onsager L (1931). Reciprocal relations in irreversible processes. I. Phys Rev.

[CR32] Petrishcheva E, Abart R (2012). Exsolution by spinodal decomposition in multicomponent mineral solutions. Acta Mater.

[CR33] Petrishcheva E, Abart R, Schäffer AK (2014). Sodium-potassium interdiffusion in potassium-rich alkali feldspar I: full diffusivity tensor at $$850^\circ$$C. Am J Sci.

[CR34] Petrishcheva E, Rieder M, Predan J (2019). Diffusion-controlled crack propagation in alkali feldspar. Phys Chem Miner.

[CR35] Petrishcheva E , Tiede L, Heuser D (2020). Multicomponent diffusion in ionic crystals: theoretical model and application to combined tracer- and interdiffusion in alkali feldspar. Phys Chem Miner.

[CR36] Petrovic R (1972) Alkali ion diffusion in alkali feldspars, Ph.D. Dissertation. Yale University

[CR37] Predan J, Kegl M, Abart R (2020). On an alternative approach for simulating chemically induced crack pattern evolutions in a single crystal. Int J Solids Struct.

[CR38] Ribbe P (1983) Chemistry, structure and nomenclature of feldspars. In: Ribbe PH (ed) Revies in mineralogy, vol 2. Mineralogical Society of America, chap 1, pp 1–19

[CR39] Rice J (1968). A path independent integral and the approximate analysis of strain concentration by notches and cracks. J Appl Mech.

[CR40] Romero I, Andres EM, Ortiz-Toranzo A (2021). Variational updates for general thermo-chemo-mechanical processes of inelastic solids. Comput Methods Appl Mech Eng.

[CR41] Schaeffer AK, Petrishcheva E, Habler G (2014). Sodium-potassium interdiffusion in potassium-rich alkali feldspar II: composition- and temperature-dependence obtained from cation exchange experiments. Am J Sci.

[CR42] Scheidl KS, Schaeffer AK, Petrishcheva E (2014). Chemically induced fracturing in alkali feldspar. Phys Chem Miner.

[CR43] Sibson R (1980). Power dissipation and stress levels on faults in the upper crust. J Geophys Res.

[CR44] Slepyan L (1993). Principle of maximum energy dissipation rate in crack dynamics. J Mech Phys Solids.

[CR45] Smith JV, Brown WL (1988). Feldspar minerals, 1 crystal structures, physical, chemical and microtextural properties.

[CR46] Sumi Y, Nemat-Nasser S, Keer L (1980). A new combined analytical and finite-element solution method for stability analysis of the growth of interacting tension cracks in brittle solids. Int J Eng Sci.

[CR47] Ulven OI, Malthe-Sorenssen A (2017) Reaction-induced fracturing: chemical-mechanical feedback. In: Heinrich W, Abart, R (ed) Mineral reaction kinetics: microstructures, textures, chemical and isotopic signatures, European Mineralogical Union Notes in Mineralogy, vol 16. p 587–615, 10.1180/EMU-notes.16.16

[CR48] Ulven OI, Jamtveit B, Malthe-Sorenssen A (2014). Reaction-driven fracturing of porous rock. J Geophys Res-Solid Earth.

[CR49] Ulven OI, Storheim H, Austrheim H (2014). Fracture initiation during volume increasing reactions in rocks and applications for co2 sequestration. Earth Planet Sci Lett.

[CR50] Verhoosel CV, Remmers JJC, Gutierrez MA (2009). A dissipation-based arc-length method for robust simulation of brittle and ductile failure. Int J Numer Meth Eng.

[CR51] Wambacq J, Ulloa J, Lombaert G (2021). A dissipation-based path-following technique for the phase-field approach to brittle and ductile fracture. Int J Numer Methods Eng.

[CR52] Wei W, Yang QS, Liang JC (2021). Theory and calculation of the mixed-mode fracture for coupled chemo-mechanical fracture mechanics. Theoret Appl Fract Mech.

[CR53] Whitney D, Broz M, Cook R (2007). Physical properties of metamorphic minerals 3. Am Miner.

[CR54] Yakobson B (1991). Morphology and rate of fracture in chemical decomposition of solids. Phys Rev Lett.

[CR55] Ziegler H (1983). Chemical reactions and the principle of maximal rate of entropy production. Z Angew Math Phys.

