# Supplementary material for: Evolution of chemically induced cracks in alkali feldspar: thermodynamic analysis
Source: Phys Chem Miner. 2022 May 3;49(5):14. doi: 10.1007/s00269-022-01183-9 (PMC9064859; doi:10.1007/s00269-022-01183-9)
Supplement: Supplementary file 1 — Supplementary file1 (DOCX 21136 kb) [file 269_2022_1183_MOESM1_ESM.pdf]

# Supplementary information

to

# Evolution of chemically induced cracks in alkali feldspar: thermodynamic analysis

by

**Rainer Abart, Elena Petrishcheva, Gerlinde Habler, Christoph Sutter, Franz-Dieter Fischer, Jozef Predan, Marko Kegl and Franz G. Rammerstorfer**

submitted to

**Physics and Chemistry of Minerals**

# 1 Experimental methods

## 1.1 Cation exchange experiments

The sanidine plates and a mixed NaCl-KCl salt with a composition of $c_{\mathrm{KCl}} = 0.25$, where $c_{\mathrm{KCl}}$ gives the mole fraction of KCl in the binary NaCl-KCl mixture, were sealed into quartz glass tubes with an inner diameter of 7 mm and 1 mm wall thickness under vacuum. The salt was applied in 40 fold excess with respect to the alkali cations contained in the feldspar plate to ensure that the salt essentially retains its original composition during cation exchange. The feldspar-salt assemblage was heated to 850°C in a box furnace for run durations of 2, 3, 5, 7, and 12 days. At 850°C the salt is molten, and the composition of alkali feldspar in equilibrium with the salt melt is $c_{\mathrm{K}} = 0.50$. After heat treatment the samples were quenched in cold water, and the quartz glass tubes were tested for tightness. The glass tubes were then opened, and the feldspar plates were retrieved by dissolving the salt using distilled water. After heat treatment the traces of parallel cracks were visible on the polished 3 x 3 mm surfaces (see Figure Sup-1).

The cation exchanged feldspar plates were embedded in cylindrical stubs of epoxy resin so that the traces of the cracks were sub vertical. The stubs were then ground until a cross section through a central position of the feldspar plate was exposed on the surface of the mount. The cross section was polished with diamond paste down to a grain size of 0.25 $\mu$m. Finally, the samples were carbon coated to ensure electrical conductivity, which is needed for scanning electron microscopy.

### 1.2 Scanning electron microscopy and electron probe micro analysis

The cross sections were investigated on an FEI Quanta 3D FEG scanning electron microscope, which was operated at an acceleration voltage of 15 kV and a beam current of 4 nA for secondary electron (SE) and back scattered electron (BSE) imaging.

The electron probe micro analyses (EPMA) were done on a Cameca SXFiveFE electron probe micro analyzer. For quantitative point analyses the instrument was operated at an acceleration voltage of 15 kV and 20 nA probe current. A defocused beam was used to prevent loss of Na. The elements Si, Al, Fe, Ca, Ba, K, Na were analyzed based on their K$\alpha$ line intensities. Natural minerals of known composition were used for standardization. Element maps for the distribution of Na and K were produced using an acceleration voltage of 10 kV, a beam current of 40 nA, a step size of 0.2 $\mu$m, and a dwell time of 40 ms. With these settings the best lateral resolution was obtained.

## 2 Gibbs energy change

As the salt melt was present in 40 fold excess with respect to the alkali cations contained in the salt and in the feldspar, the composition of the salt remained essentially constant during the exchange. The cation exchange may be described by the stoichiometric equation

$$\mathrm{Na_{0.15}K_{0.85}AlSi_3O_8} + 0.35\ \mathrm{NaCl} = \mathrm{Na_{0.5}K_{0.5}AlSi_3O_8} + 0.35\ \mathrm{KCl},$$

where $\mathrm{Na_{0.15}K_{0.85}AlSi_3O_8}$ represents the original feldspar, and $\mathrm{Na_{0.5}K_{0.5}AlSi_3O_8}$ corresponds to the feldspar in equilibrium with the NaCl-KCl salt melt. The molar Gibbs energy of reaction was calculated based on the thermodynamic data from Holland and Powell (2011) for the apparent molar Gibbs energies of the Na and K end-member components of the alkali-feldspar solid-solution and the binary NaCl-KCl salt melt. For the alkali feldspar an asymmetric two parameter mixing model employing the Margules parameters determined for Volkesfeld Sanidine by Neusser et al (2012) was applied, and an ideal molecular mixing model was adopted for the salt melt. From this, the molar Gibbs energy of reaction was obtained as $\Delta_r g = -5015$ J/mol. Given the molar volume $\bar{\Omega} = 1.084 \cdot 10^{-4}$ m$^3$/mol of alkali feldspar with $c = 0.67$ and a volume of the simulation cell of $\Omega = 0.028 \cdot 0.5 \cdot 1 \cdot 10^{-9}\mathrm{m}^3$, this corresponds to a free energy change of $-6.5 \cdot 10^{-4}$ J for the full equilibration of the feldspar contained in the simulation cell with the salt melt.

It must be noted, that some of this energy is stored in the surface energy of the newly formed crack flanks. Given that two cracks are contained in the simulation cell the surface area of the four corresponding crack flanks is calculated as $5 \cdot 10^{-4}\mathrm{m} \cdot 10^{-3}\mathrm{m} \cdot 4 = 2 \cdot 10^{-6}\mathrm{m}^2$ for the case, when the cracks extend through the entire simulation cell in the $y$ and $z$ directions. The crack surface energy was estimated to $\leq$1 J/m$^2$ by Petrishcheva et al (2019) so that the total surface energy associated with the cracks in the simulation cell is $\leq 2 \cdot 10^{-6}$J.

This is $\leq 0.3\%$ of the free energy change due to chemical equilibration between the feldspar in the simulation cell and the salt melt, which corresponds to a minute reduction of the total free energy change. Thus, the total free energy available for dissipation in the volume $\Omega$ of the simulation cell is $-6.5 \cdot 10^{-4}$ J.

# 3 Strain state

In a small displacement setting, the strain tensor is given as

$$\varepsilon_{ij} = \frac{1}{2}(\partial_i u_j + \partial_j u_i)$$

and is composed of the eigenstrain tensor and the elastic strain tensor. The eigenstrain state is obtained from the calculated concentration field, using the compositional dependence of the lattice parameters of alkali feldspar (Kroll et al, 1986) following the procedure described by Scheidl et al (2014). For monoclinic alkali feldspar the chemically induced eigenstrain tensor $\boldsymbol{\varepsilon}_{ij}^{\text{chem}}$ reads

$$\boldsymbol{\varepsilon}_{ij}^{\text{chem}} = \begin{pmatrix} \varepsilon_{11}^{\text{chem}} & 0 & \varepsilon_{13}^{\text{chem}} \\ 0 & \varepsilon_{22}^{\text{chem}} & 0 \\ \varepsilon_{13}^{\text{chem}} & 0 & \varepsilon_{33}^{\text{chem}} \end{pmatrix}.$$

Based on the lattice parameters given by Kroll et al (1986) for the compositions $c = 0.84$ and $c = 0.69$, the components $\hat{\varepsilon}_{11} = -7.6 \times 10^{-3}$, $\hat{\varepsilon}_{22} = -5.1 \times 10^{-4}$, $\hat{\varepsilon}_{33} = -5.9 \times 10^{-4}$, $\hat{\varepsilon}_{13} = -1.9 \times 10^{-3}$ are obtained for a compositional shift from $c = 0.84$ to $c = 0.69$, $\Delta c = -0.15$. The quantity $\Delta c = -0.15$ is considered as a reference compositional shift. The tensor $\hat{\boldsymbol{\varepsilon}}_{ij}$ is the corresponding eigenstrain tensor (Scheidl et al, 2014). In the compositional range of interest, the lattice parameters of alkali feldspar show a linear dependence on composition (Kroll et al, 1986). The chemically induced eigenstrain state corresponding to a compositional shift from the original composition $c = 0.85$ to some new composition $c$ is obtained as

$$\varepsilon_{ij}^{\text{chem}} = \frac{c - 0.85}{-0.15}\,\hat{\varepsilon}_{ij}.$$

# 4 Stress-state

The domain of interest is defined as a planar cell, which is fixed at the left bottom corner and can move only in $x$-direction at the right bottom corner. Periodic boundary conditions are assumed at both sides. Generalized plane strain for the 2D-configuration is applied, implying that the displacement of the whole simulation cell in $z$-direction assumes the same value in all points, with no resultant force in $z$-direction on the cell.

The elasticity tensor is formulated with respect to the global $x, y, z$-system. In the according tensor/vector notation $x$, $y$, $z$ are assigned to 1, 2, 3, respectively. The elasticity tensor is arranged with the data from Haussuehl (1993)

as a $6 \times 6$ tensor relating the stress vector $\boldsymbol{\sigma}^T = (\sigma_{11}, \sigma_{22}, \sigma_{33}, \sigma_{12}, \sigma_{13}, \sigma_{23})$ to the strain vector $\boldsymbol{\epsilon}^T = (\epsilon_{11}, \epsilon_{22}, \epsilon_{33}, \epsilon_{12}, \epsilon_{13}, \epsilon_{23})$. About $10^5$ linear 4-node elements are applied with a dense mesh near the crack tips and along the crack and a less dense mesh along the side flanks of the simulation cell.

# 5 J-integral

For the given geometry and for the case of zero eigenstrain, the $J$-integral reads (Cherepanov, 1967; Rice, 1968)

$$J = \int_\Gamma \Big( W dy - \sum_{i,j=1}^{2} \sigma_{ij} n_i \partial_x u_j ds \Big). \tag{1}$$

$W(x,y)$ is the strain energy density, $\sigma_{ij}(x,y)$ is the stress tensor, $u_j(x,y)$ is the displacement vector, $n(x,y)$ is the normal vector to the integration path $\Gamma$, and $ds = |d\boldsymbol{s}|$ is the length of an infinitesimal tangent vector along $\Gamma$. For external loading of a homogeneous material, free of eigenstrain, the J-integral is path-independent (Cherepanov, 1967; Rice, 1968). However, if an eigenstrain state (as in the current system) exists, the J-integral is path-dependent (Simha et al, 2005). A reasonable estimate of the J-integral, representing the energy release rate of crack propagation can be obtained, when the smallest numerically feasible path around the crack tip is used for integration (Simha et al, 2003).

The actual eigenstrain state is related to the composition field via the compositionally dependent lattice parameters of the alkali feldspar. We solve the diffusion problem, fully coupled with the mechanical problem, numerically following the approach of Predan et al (2020). The J-integral is evaluated using the strain and stress state due to the eigenstrain state obtained from the calculated concentration field.

# 6 Local control on crack propagation path

If we assume that the direction of an increment of crack growth is determined by incremental maximization of the rate of the total free energy dissipation during this growth increment, we need to consider the following relations: The rate of the chemical dissipation is determined by integrating the square of the diffusive flux over the entire actual volume $\Omega$. Its variations due to a variation of the orientation of the growth increment are small as compared to its actual value. In computational simulations, this leads to the so-called "problem of small differences between large numbers". The mechanical dissipation results from what happens directly at the crack tip due to the formation of new surfaces during the growth increment. The mechanical dissipation rate depends on the local stress state at the crack tip in conjunction with the - nota bene - orientation dependent critical energy release rate. Assuming linear elastic material behavior, the stress state depends on the strain state and

the - nota bene - anisotropic elasticity tensor, which, for the sake of simplicity, is assumed to be independent of the feldspar composition. The total strain state stems from the eigenstrains in combination with their obstructions by the boundary conditions including the free surfaces given by the current configuration. Concluding, the eigenstrain tensor depends on the local concentrations of the cations (Kroll et al, 1986). The concentration field is a result of the transient diffusion process, which depends on the anisotropic diffusivity tensor (Petrishcheva et al, 2014) and the boundary conditions given by the current configuration. In conclusion, we are confronted with a complex, highly coupled, transient multi-physics process. A computational simulation of this process has been presented by Predan et al (2020) for straight cracks growing perpendicular to the specimen surface. Computationally simulating the development of the experimentally observed curved or "oscillating" cracks is beyond the scope of this study. However, a simplified narrative description of the development of such oscillating cracks is given. We define the direction normal to the specimen surface as "vertical" and the direction parallel to it as "horizontal". We disregard the comparatively small contribution due to the variation of the chemical diffusion with a change in the direction of incremental crack growth.

The following facts should be noted:

1. The diffusion coefficient in the horizontal direction is about one order of magnitude larger than that in the vertical direction (Petrishcheva et al, 2014). As a consequence, diffusion from a close to vertical portion of a crack is more efficient than from a (sub)horizontal portion.

2. During the initial growth period of straight parallel cracks the dissipation rate goes through a minimum.

3. For a given shift towards more Na-rich compositions, the absolute value of the eigenstrain component in horizontal direction is by a factor of about five larger than the one in vertical direction. All eigenstrain components resulting from progressive in-diffusion of Na have negative values (Predan et al, 2020). The eigenstrains have a tendency to produce tensile stresses, which are significantly larger in the horizontal direction than in the vertical one. From this fact, one may conclude that cracks should predominantly grow in vertical direction.

4. The horizontal direction corresponds to a prominent cleavage plane. Therefore, the critical energy release rate for horizontal cracks is supposedly significantly lower than for vertical cracks. Thus, even small tensile stresses in vertical direction may lead to crack growth in horizontal direction.

We now consider the growth of a crack, which nucleated eccentrically between two pre-existing straight cracks. We assume that it starts to grow close to its right neighbour. Immediately after the diffusion process started, the Na concentration attains the highest possible level at the specimen surface, and an extreme vertical concentration gradient exists immediately beneath the specimen surface providing remarkable horizontal tensile stresses at the specimen surface. Due to the stress boundary conditions at the free surface, no vertical

stresses exist in this region. Thus, all cracks start in vertical direction, which is generally observed indeed.

With successive crack growth the free-surface boundary conditions along the newly formed crack flanks imply that the horizontal tensile stresses diminish towards the vertical crack. Because of the short distance between the considered short crack and the pre-existing right neighbour crack, these boundary conditions together with the release of the horizontal tensile stresses due to opening of the cracks prevents the area around the short crack from the development of significant horizontal tensile stresses. Consequently, even small vertical tensile stresses associated with the concentration gradients generated by diffusion perpendicular to the vertical crack flanks in combination with the small value of the critical energy release rate for crack growth in horizontal direction induce the initially vertical crack to turn away from the nearby vertical neighbour crack and into a sub-horizontal direction. When the stress release effect diminishes and horizontal tensile stresses resulting from diffusion perpendicular to the meanwhile subhorizontal crack flanks start to prevail again, the crack turns towards the vertical direction again. This happens even though the critical energy release rate is larger in this direction. However, the more the crack turns to the vertical direction, the more the horizontal tensile stresses are released. As a consequence, the considered curved crack turns again towards a more horizontal direction.

With the progress of diffusion perpendicular to the flanks of the straight neighbouring cracks, which meanwhile have grown significantly longer, and the release of horizontal tensile stress close to these cracks by their opening, the stress state at the tip of the considered short crack becomes more and more dominated by the horizontal tensile stresses. They have their maximum mid way between the long vertical neighbouring cracks. Thus, the back and forth turning is “damped out”, and the tip of the intermediate crack finds its position approximately in the middle between its neighbours and moves in vertical direction. The considered crack has now achieved a depth at which the position of the crack in the middle between the two neighbouring cracks ensures a maximum rate of free energy dissipation.

The history of the path along which the tip of the intermediate crack migrates, starting from an eccentric position at the specimen surface up to the mid-distance position with final growth in vertical direction, is initially controlled by fracture and then more and more by diffusion.

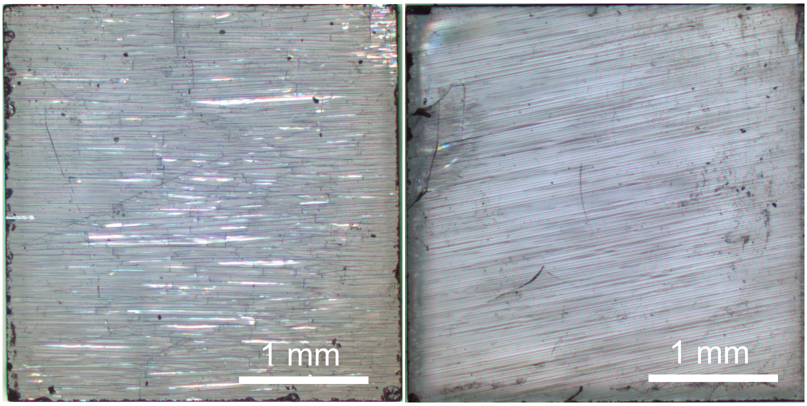


**Fig. Sup-1** Reflected light foto micrograph of the traces of cracks on the 3 x 3 mm sized (001) (left) and (010) (right) surfaces of the cuboid plates after exchange for 5 days.

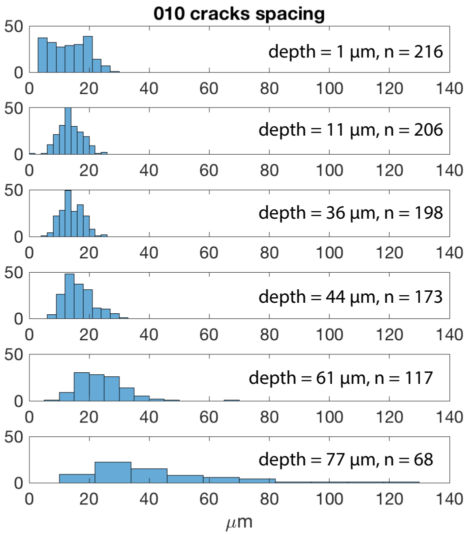


**Fig. Sup-2** Spacing of cracks emanating from the polished (010) surface from 7 days experiment at different depth below the sample surface

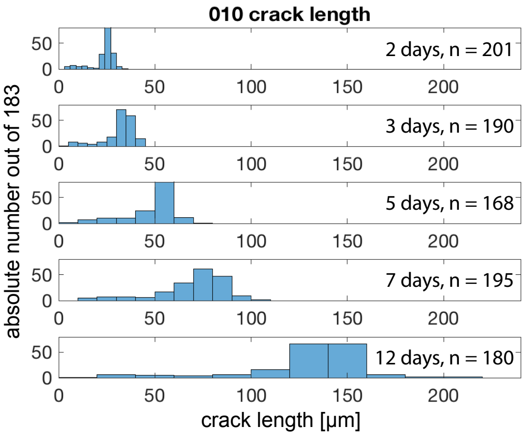


**Fig. Sup-3** Length of cracks emanating from the polished (010) surface after cation exchange for 2, 3, 5, 7, and 12 days

# References

Cherepanov G (1967) Crack propagation in continuous media. Journal of Applied Mathematics and Mechanics-USSR 31(3):503+. https://doi.org/{10.1016/0021-8928(67)90034-2}

Haussuehl S (1993) Thermoelastic properties of beryl, topaz, diaspore, sanidine and periclase. Zeitschrift für Kristallographie 204:67–76

Holland TJB, Powell R (2011) An improved and extended internally consistent thermodynamic dataset for phases of petrological interest, involving a new equation of state for solids. Journal of Metamorphic Geology 29(3):333–383. https://doi.org/{10.1111/j.1525-1314.2010.00923.x}

Kroll H, Schmiemann I, von Coelln G (1986) Alkali feldspar solid-solutions. American Mineralogist 71:1–16

Neusser G, Abart R, Fischer FD, et al (2012) Experimental Na/K exchange between alkali feldspar and an NaCl-KCl salt melt: chemically induced fracturing and element partitioning. Contributions to Mineralogy and Petrology 164(2):341–358. https://doi.org/{10.1007/s00410-012-0741-9}

Petrishcheva E, Abart R, Schäffer AK, et al (2014) Sodium-potassium interdiffusion in potassium-rich alkali feldspar I: Full diffusivity tensor at 850°C. American Journal of Science 314(9):1284–1299

Petrishcheva E, Abart R, Schaeffer AK, et al (2014) Sodium-potassium interdiffusion in potassium-rich alkali feldspar I: full diffusivity tensor at 850 degrees C. American Journal of Science 314(9):1284–1299. https://doi.org/

{10.2475/09.2014.02}

Petrishcheva E, Rieder M, Predan J, et al (2019) Diffusion-controlled crack propagation in alkali feldspar. Physics and Chemistry of Minerals 46:15–26. https://doi.org/{10.1007/s00269-018-0983-9}

Predan J, Kegl M, Abart R, et al (2020) On an alternative approach for simulating chemically induced crack pattern evolutions in a single crystal. International Journal of Solids and Structures 202:575–586. https://doi.org/{10.1016/j.ijsolstr.2020.06.006}

Rice J (1968) A Path Independent Integral and the Approximate Analysis of Strain Concentration by Notches and Cracks. Journal of Applied Mechanics 35(2):379–386. https://doi.org/{10.1115/1.3601206}

Scheidl KS, Schaeffer AK, Petrishcheva E, et al (2014) Chemically induced fracturing in alkali feldspar. Physics and Chemistry of Minerals 41(1):1–16. https://doi.org/{10.1007/s00269-013-0617-1}

Simha N, Fischer FD, Kolednik O, et al (2003) Inhomogeneity effects on the crack driving force in elastic and elastic-plastic materials. Journal of the Mechanics and Physics of Solids 51:209–240

Simha N, Kolednik O, Fischer FD (2005) Material force models for crack – influences of eigenstrains, thermal strains & residual stresses. Proc 11th Int Conf on Fracture Topic 02:417–422
